# Effects of intranasal insulin application on the hypothalamic BOLD response to glucose ingestion

**DOI:** 10.1038/s41598-017-13818-x

**Published:** 2017-10-17

**Authors:** Anna M. van Opstal, Abimbola A. Akintola, Marjan van der Elst, Rudi G. Westendorp, Hanno Pijl, Diana van Heemst, Jeroen van der Grond

**Affiliations:** 1 Department of Radiology, Leiden University Medical Centre, Leiden, The Netherlands; 2 Department of Internal Medicine, Section Gerontology and Geriatrics, Leiden University Medical Centre, Leiden, The Netherlands; 3 Department of Public Health and Centre for Healthy Ageing, University of Copenhagen, Copenhagen, Denmark; 4 Department of Internal Medicine, Section Endocrinology, Leiden University Medical Centre, Leiden, The Netherlands

## Abstract

The hypothalamus is a crucial structure in the brain that responds to metabolic cues and regulates energy homeostasis. Patients with type 2 diabetes demonstrate a lack of hypothalamic neuronal response after glucose ingestion, which is suggested to be an underlying cause of the disease. In this study, we assessed whether intranasal insulin can be used to enhance neuronal hypothalamic responses to glucose ingestion. In a randomized, double-blinded, placebo-controlled 4-double cross-over experiment, hypothalamic activation was measured in young non- diabetic subjects by determining blood-oxygen-level dependent MRI signals over 30 minutes before and after ingestion of 75 g glucose dissolved in 300 ml water, under intranasal insulin or placebo condition. Glucose ingestion under placebo condition lead to an average 1.4% hypothalamic BOLD decrease, under insulin condition the average response to glucose was a 2.2% decrease. Administration of water did not affect the hypothalamic BOLD responses. Intranasal insulin did not change circulating glucose and insulin levels. Still, circulating glucose levels showed a significant dampening effect on the BOLD response and insulin levels a significant strengthening effect. Our data provide proof of concept for future experiments testing the potential of intranasal application of insulin to ameliorate defective homeostatic control in patients with type 2 diabetes.

## Introduction

Worldwide, there has been a rapid increase in the number of patients who have type 2 diabetes mellitus. Type 2 diabetes, found in >90% of all cases of diabetes, is characterized by insulin resistance and a defective secretion of insulin. For several decades, glucose homeostasis was assumed to be regulated primarily via peripheral mechanisms and centered around insulin produced by pancreatic islet cells and its effects on suppression of hepatic glucose production and, stimulation of glucose uptake by adipose tissue and muscle^1–3^. Recent data however demonstrated that the brain also plays a pivotal role in glucose regulation^4,5^. A key brain area that is involved in glucose homeostasis is the hypothalamus, which regulates metabolic homeostasis via its reciprocal projections to other brain regions and the periphery^6^. The hypothalamus senses, integrates and co-ordinates circulating metabolic cues, thereby aiding regulation of body weight and feeding behavior^6,7^. Insulin, a signal reflecting energy availability and reserves, provides direct negative feedback to hypothalamic nuclei that control energy and glucose homeostasis^8^. Previous studies have clearly shown that insulin receptors and insulin signaling in the hypothalamus is vital in the regulation on glucose and energy metabolism^8–11^. Studies on the role of the brain in glucose regulation in humans have been facilitated by non-invasive techniques such as measurement of blood oxygen level dependent (BOLD) brain responses, which allows quantification of brain activities over time during functional MRI studies. BOLD MRI contrast is a reflection of hemodynamic changes associated with neuronal activity due to changes in local concentrations of oxygenated and deoxygenated hemoglobin^12,13^. These BOLD changes can be measured in the hypothalamus, in response to glucose or to other stimuli^14^. In healthy men, a prolonged, dose-dependent decrease in hypothalamic BOLD signal was observed in response to glucose ingestion, showing adaptability of the hypothalamus to nutrient ingestion^14,15^. Conversely, in patients with type 2 diabetes, a lack of significant decrease in BOLD signal was observed in response to glucose ingestion, implying diabetes- induced failure to inhibit hypothalamic neuronal activity in response to glucose ingestion^16^. Interestingly, in patients with type 2 diabetes, this absent hypothalamic response normalized after a 4-day very-low-calorie diet, indicating that hypothalamic dysfunction to glucose sensing is potentially reversible^17^.

We hypothesize that deficits in central insulin action play a role in defective hypothalamic gluco-regulatory responses and that increasing central insulin levels may potentiate brain responses to glucose. Earlier studies have shown that insulin, and other compounds, can be non-invasively delivered to the CSF and cross the brain barrier via the intranasal route, without spilling over to the periphery, allowing for a specific increase in central insulin levels^18,19^. Several studies have shown that intranasal insulin has an effect on various hypothalamic functions, indicating that the hypothalamus is a target for and is reached by intranasal aplicaition of insulin^20,21^. In a randomized, double blind, placebo-controlled cross-over experiment, we aimed to investigate the hypothesis that intranasal insulin application may potentiate the BOLD response to glucose ingestion in the hypothalamus in young healthy volunteers. First, we applied insulin or placebo using a customized nasal atomizer. Thereafter hypothalamic BOLD signals were measured for 30 minutes before, during and  after ingestion of 75 g glucose dissolved in water, or plain water being the control condition.

## Results

### Subject characteristics

The characteristics of study subjects are summarised in Table 1. The participants had a mean age of 22 years and mean BMI of 23.6 kg/m^2^. The mean fasted glucose was 4.8 mmol/L, median insulin level was 0.84 µU/ml and the median HOMA-IR index was 1.2.

**Table 1:** Characteristics of study subjects.

	**Characteristics**
**Demographics**	N = 8
Age in years (SD)	22.3 (1.8)
BMI in kg/m^2^	23.6 (2.2)
Alcohol intake (units/week)	14.4 (2.2)
Systolic BP (mmHg)	125 (9.3)
Diastolic BP (mmHg)	74 (7.2)
**Metabolic**	
Fasted glucose in mmol/L	4.8 (0.3)
Fasted insulin in µU/ml, median (IQR)	0.84 (0.69, 0.95)
HOMA-IR index, median (IQR)	1.2 (1.0, 1.5)

Unless otherwise stated, values are means (standard deviation). BMI: body mass index.

### Blood glucose and insulin trajectories after intranasal insulin application

**Figure 1:**
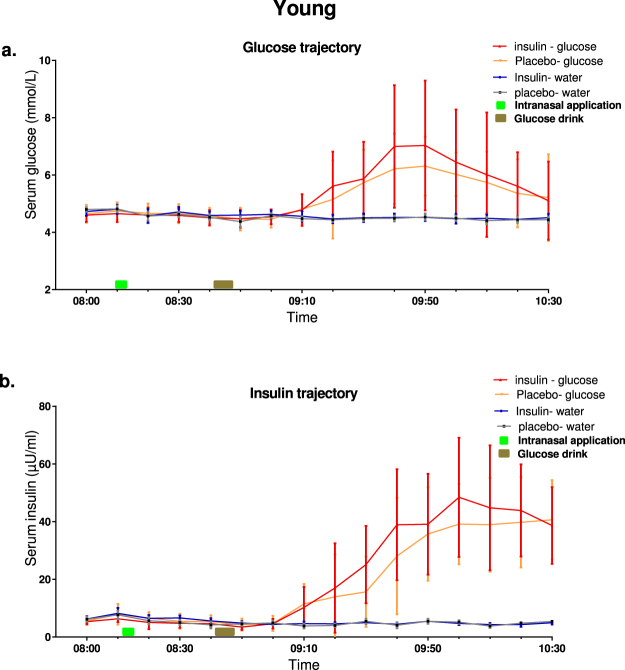
Glucose and insulin trajectories during the experimental period. Concentration of (**a**) glucose and (**b**) insulin in serum before and after intranasal insulin application (40 IU Insulin actrapid, blue line) or placebo (saline, red line) using Vianase nasal atomizer. Data are presented as mean (Standard error) of A. glucose and B. insulin levels measured every 10 minutes. The green bar represents the timing of intranasal application, with the brown bar represent the timing of ingestion of a glucose drink or water during the fMRI scanning.

The mean serum glucose and insulin trajectories of all participants over 2.5 hour encompassing the time period before, during and after the intranasal application of placebo or insulin and MRI scanning procedure are shown in Fig. 1. Serum glucose levels after drinking water were stable throughout the experimental period and were not significantly different between insulin and placebo conditions (Fig. 1a). After glucose ingestion, the serum glucose levels increased in both intranasal insulin and placebo conditions, with the rise in glucose levels starting approximately 15 minutes after glucose ingestion. A peak glucose level of 7.0 mmol/l was reached 50 minutes after glucose ingestion under the intranasal insulin condition. For the placebo condition, a peak of 6.3 mmol/l was reached 60 minutes after glucose ingestion. Thereafter, glucose levels for both insulin and placebo conditions declined to 5.2 mmol/l at the end of the experinment.

Serum insulin levels after drinking water were stable throughout the experimental period and were not significantly different between the intranasal insulin and placebo conditions (Fig. 1b). After glucose ingestion serum insulin levels increased under both intranasal insulin and placebo conditions. After intransal insulin application, the serum insulin levels reached a peak of 48.4mU/L at 80 minutes after glucose ingestion. Under intransal application of placebo, a peak in serum insulin of 40.65 mU/L was reached 90 minutes after ingesting the glucose drink.

### Changes in hypothalamic BOLD response

**Figure 2:**
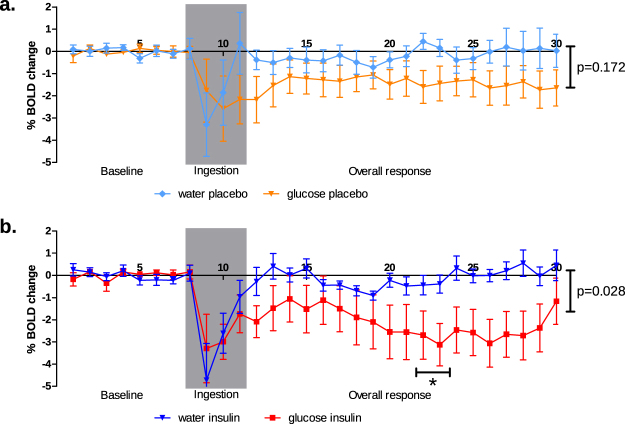
Hypothalamic BOLD responses under placebo and insulin conditions. Baseline BOLD signal measured from minute 1 to 8, ingestion of either glucose solution or water from minute 8 through 11 (indicated by grey box), overall response period after ingestion of glucose solution or water from minute 12 through 30. Panel a: light blue line: response to water ingestion under placebo condition, orange line: response to glucose ingestion under placebo condition. Panel b: dark blue line: response to water ingestion under insulin condition, red line: response to glucose ingestion under insulin condition. *Indicates significant differences at p < 0.05 Bonferroni correct for multiple comparison for separate time window analysis.

Figure 2 shows the group averaged hypothalamic BOLD responses before, during and after ingestion of study stimulus (glucose or water). The water and glucose responses under placebo condition are shown in panel a. The corresponding responses under insulin condition are shown in panel b.

Over the entire 22- minute post- drinking period, the BOLD response after water ingestion under placebo condition, was 0.2% lower compared with baseline, and after glucose ingestion 1.4% lower compared with baseline. Although the response was lowered after ingestion of glucose compared to water ingestion no significant difference between the two conditions was found. Also, no differences between these conditions for the two minute time intervals were found.

Panel B shows the water and glucose responses under insulin condition. Over the entire period, the BOLD response after water ingestion was 0.1% lower compared with baseline, and after glucose ingestion 2.2% lower compared with baseline. For the entire post drink period, the glucose BOLD response was significantly (p = 0.028, whole time series mixed-model analysis) lower than the water response. Especially between 20 and 23 minutes after ingestion this difference becomes most prominent (p < 0.05 Bonferroni corrected, 2-min window mixed model analysis).

### Effects of plasma glucose and insulin levels on the BOLD response

Circulating glucose levels showed a significant effect on the BOLD response (estimated effect: +0.3% BOLD change, p = 0.001). Indicating that a higher plasma glucose level leads to a dampened decrease in the BOLD signal of the hypothalamus. Circulating plasma insulin levels had a significant, albeit small, decreasing effect on the BOLD response (estimated effect: −0.03% BOLD change, p = 0.005). The HOMA-IR index did not have a significant effect on the BOLD response.

## Conclusions

To study the effects of intranasal insulin application on neuronal activity in the hypothalamus after glucose ingestion in young healthy volunteers, we performed a randomized, double blind, placebo-controlled cross-over proof of concept experiment and applied insulin or placebo via the nose using a customized nasal atomizer. The majornew finding of our study is that the decrease in fMRI BOLD response in the hypothalamus after glucose ingestionwas augmented after intranasal application of insulin. Furthermore, we found that circulating glucose and insulin levels did not change after intranasal application of insulin compared to placebo indicating that there was no spill over to the systemic circulation and we were able to target the CNS specifically. Nevertheless, in line with the absence of reduction in hypothalamic BOLD response to glucose previously observed in patients with diabetes type 2 with increased circulating glucose^16,17^, we found that in healthy volunteers higher plasma glucose level also lead to reduced hypothalamic BOLD responses.

The hypothalamus plays a key role in the central regulation of food intake and energy metabolism, and specific hypothalamic nuclei are critical for the control of peripheral metabolism by insulin^6,8^. We found intranasal insulin administration augmented the decrease in hypothalamic BOLD response to glucose ingestion, with an average decrease in the BOLD signal of 1.2% after intranasal placebo applications and 2.2% when the glucose ingestion was preceded by intranasal application of insulin. Indicating that central insulin can affect the hypothalamic function to maintain glucose homeostasis as is suggested by earlier studies^8,10,22^. Among patients with type 2 diabetes, the glucose-induced reduction in hypothalamic BOLD signal was absent, suggesting that glucose ingestion failed to inhibit hypothalamic neuronal activity^16,17^. Considering that we found the decreased BOLD signal to ingested glucose was potentiated after intranasal insulin, future experiments might be aimed at testing the potential of intranasally applied insulin to rescue the defective BOLD response that was observed in diabetic patients. Future studies could also be targeted at performing these experiments under conditions of increased adiposity, such as obesity, which has also been implicated to have effects on the hypothalamic response^23^.

One limitation of this study is that it was done only in male volunteers and it can be speculated that a sex dimorphism is present since it is known that there are several sex-specific differences in energy metabolism^24^. This decreases the generalizability of our findings. Another limitation is the indirect measurement of neural activity in the targeted brain area using BOLD measurement technique, as it is unclear what direct functional consequences these signals reflect without additional measurements of downstream functional effects. Furthermore, other pathways could also affect the hypothalamic response to glucose besides the direct sensing of glucose by the hypothalamus. Other studies have shown that dopaminergic pathways also respond to energy ingestion and could thus have an effect on the hypothalamic response^25^. To determine this interplay BOLD measurements in the limbic system might thus be useful in future studies. The strength of this study is the methodological rigor and the fact that the participants were their own controls, thus ruling out possible dilution of effects that could have been introduced due to differences in individual phenotypes.

Summarily, using a randomized, double blind, placebo-controlled cross- over proof of concept experiment, we studied the effects of intranasal insulin application on blood oxygen level dependent (BOLD) signals as proxy for neuronal activity in the key brain area of the hypothalamus that has been implicated in responses to metabolic cues. We found that intranasal insulin augmented the decrease in fMRI BOLD signal in response to glucose ingestion in the hypothalamus. Further research is needed to investigate whether intranasal application of insulin has similar effects on hypothalamic BOLD responses in patients with type 2 diabetes, and what the functional consequences are.

## Methods

### Ethics statement

The study was approved by the Medical Ethical Committee of Leiden University Medical Centre under protocol P13.164 and the Dutch competent authority (Centrale Commissie Mensgebonden Onderzoek (CCMO)) under protocol code number NL45043.058.13. The study is registered in the European Clinical Trials Database under number 2012-005650-29 on the 9^th^ of April 2014. All investigations have been conducted according to the principles expressed in the Declaration of Helsinki. All participants provided written informed consent after complete written and verbal description of the study was given.

### Experimental subjects

Our study population consisted of 8 healthy, normal weight (20 kg/m^2^ < BMI < 25 kg/m^2^) adult male volunteers from the general population. Exclusion criteria included fasting plasma glucose above 7 mmol/l, anaemia (haemoglobin < 7.1 mmol/l), any significant endocrine, neurological and cardiovascular diseases, or use of medication known to influence lipolysis, thyroid function, glucose metabolism, GH/IGF-1 secretion or any other hormonal axis. Furthermore, persons with a history of smoking addiction, alcohol addiction or substance abuse were excluded as well as persons with characteristics that might interfere with effective intranasal application, including presence of anatomic deviations of the nose or chronic colds, running nose or allergies.

### Experimental protocol

We performed a randomized, double-blinded, placebo-controlled cross- over experiment. For each participant, the study was carried out on four study visits, each spaced apart by one week. The participants were randomized over two treatment arms, with half of the participants having intranasal application of placebo first and the other half intranasal application of insulin first. Concealment of treatment allocation was ensured by delivery of experimental medication in same type of vial that was prepared and re-labelled by the hospital pharmacy. De-blinding was done at the end of the study. After a 10 hour overnight fast, participants arrived at the MRI facilities at 8AM. First, a baseline venous blood sample was collected in a serum-separator (SST)-tube and thereafter, every 10 minutes, 4 ml of blood was collected into a SST-tube and 2 ml into K3-EDTA tube. Ten minutes after the first blood withdrawal, the study intervention was applied, which consisted of intranasal application of 40 IU insulin (Actrapid; Novo Nordisk, Mainz, Germany) or placebo (Normal saline), applied using the ViaNase Electronic Atomizer (Kurve Technology inc) 20 minutes before the start of the MRI scanning procedure. At 30 minutes after application of intranasal insulin/placebo, single slice BOLD mid-brain fMRI scans were performed, during which participants drank either the glucose solution or water (75 gr of glucose dissolved in 300 ml of tap water or 300 ml of plain tap water). This timing of ingestion of the glucose and/or water stimulus was chosen as we expected the greatest effect of the intranasal treatment at this time. As previous studies have shown that it takes 30 minutes for insulin to reach maximal concentrations in the CSF^26^, and a similar time window has been used previously in other studies where brain – related effects of intranasal insulin were studied^21,27,28^. The total time for the BOLD scans (baseline measurement, ingestion and post-ingestion measurements) was 30 minutes.

### Processing of blood samples

After blood withdrawal, K3-EDTA tubes were immediately placed on ice before centrifugation. Serum tubes were kept at room temperature and centrifuged at 3520 RPM at 4 °C for 10 minutes when the samples were clotted, usually between 30–60 minutes. EDTA plasma and serum samples were stored in two aliquots of 500 µl during the rest of the study day at −20 °C. After completion of sample collection, these were stored at −80 °C. All laboratory measurements were performed with fully automated equipment and diagnostics from Roche Diagnostics (Almere, The Netherlands). Glucose levels were measured using Hitachi Modular P800 from Roche (Almere, the Netherlands), with coefficient of variation (CV) for measurement less than 1%. Insulin levels were measured using the Immulite 2500 from DPC (Los Angeles, CA), with CV less than 6%. HOMA-IR index was calculated according to the formula: fasting insulin × fasting glucose/22.5.

### MRI acquisition and analysis

**Figure 3:**
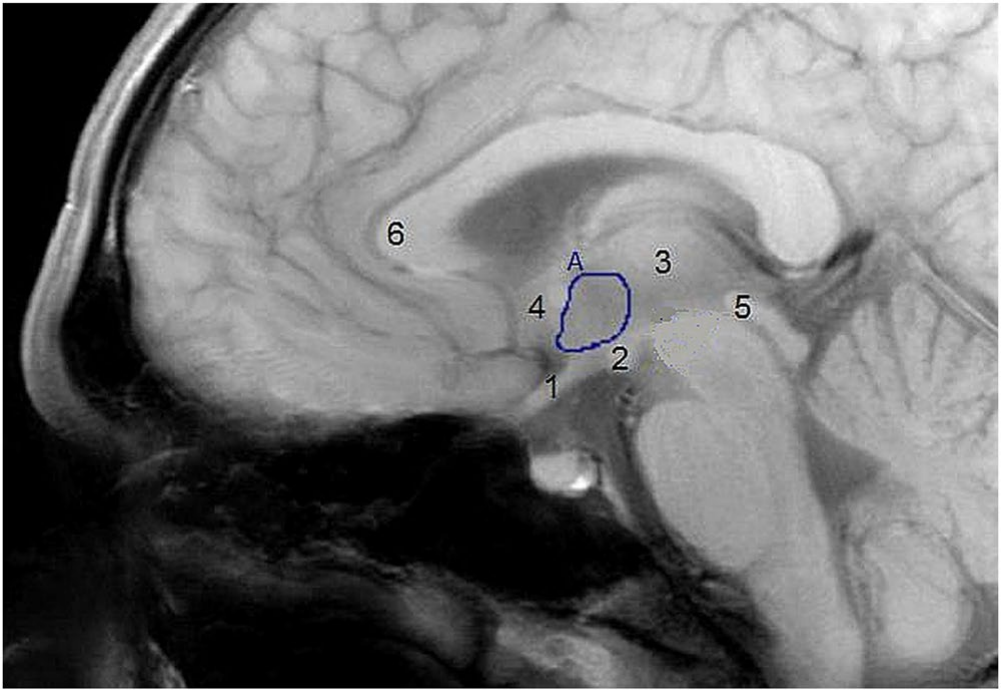
Segementation of the hypothalamus region of interest (ROI). For the hypothalamus ROI (A), the optic chiasm (1), the mammillary bodies (2), the thalamus (3) the anterior commissure (4) and the top of the cerebral aqueduct (5) were used as landmarks. The reference ROI was drawn superior of the genu to the corpus callosum (6) in the grey matter.

MRI was performed on a 3.0 Tesla clinical MRI scanner (Achieva Philips Healthcare, Best, the Netherlands). We performed a structural mid brain T1 scan (single slice scan, repetition time 550 ms, echo time 10 ms, field of view 208 × 208 mm, voxel size = 0.52 × 0.52 × 14 mm, scan time 1.14 min) and a high resolution mid brain single slice fMRI scan (repetition time 120 ms, echo time 30 ms, field of view 208 × 208 mm, voxel size = 0.81 × 0.81 × 14 mm, scan time 30 min, 700 dynamic volumes). Both of these single slice mid-sagittal scans were planned using third ventricle, the corpus callosum and brain midline as landmarks. Data was pre-processed as described in previous studies^17^. Data was averaged for each set of 4 subsequent volumes, reducing the 700 dynamic scans to 175. The hypothalamus was segmented manually, using the mid brain single slice anatomical image as a guide for delineation. Manual segmentation was performed on the middle dynamic volume of the fMRI scan as described previously using the anterior commissure, the optic chiasm and the mammillary bodies as anatomical landmarks^14,17^. Example delineation of the hypothalamus, with the anatomical landmarks used for delineation, is shown in Fig. 3. To correct for scanner drift, all hypothalamic BOLD values were corrected using an internal reference ROI in the grey matter superior of the genu of the corpus callosum.

### Statistical Analysis

For statistical analyses, Statistical Package for Social Sciences (SPSS) software for windows (version 22.0) was used. Graphs were made using GraphPad Prism version 5 (GraphPad, San Diego, CA). Descriptive statistics were used to summarise the characteristics of both study groups. All fMRI results are reported as percentage BOLD change, relative to baseline; 0–8 minute BOLD response before drinking of the test solution. For presentation purposes, the individual 175 dynamic scans time points were pooled into ‘per minute’ data points: The first 8 minutes were considered baseline BOLD response. Data from minute 8 through 11 were omitted from the analysis due to non-physiologic excessive BOLD changes caused by swallowing of the drink. Statistical analysis of the differences in effect between the study stimuli on the overall BOLD response was performed by a single linear mixed model analysis using the study stimulus as a fixed effect, time point as a covariate and subject per occasion as a random factor using the total time series. Differences in treatment effect was deemed significant at p < 0.05. To test for treatment effects over time the same model was used per two minute intervals instead of the entire series. P-values for the analysis per two minute intervals are reported corrected for multiple comparison with a Bonferonni correction of eight (p < 0.00625). To test for the influence of blood glucose and insulin levels and the HOMA-IR index on the response to the study stimuli, additional mixed model analyses were performed, in which these values were added as covariates. These effects of glucose and insulin on the BOLD response are reported as estimated effects in percentage BOLD change.

### Data availability

The datasets generated during and/or analysed during the current study are available from the corresponding author on reasonable request.
